# Timing Is Everything—The High Sensitivity of Avian Satellite Cells to Thermal Conditions During Embryonic and Posthatch Periods

**DOI:** 10.3389/fphys.2020.00235

**Published:** 2020-03-31

**Authors:** Orna Halevy

**Affiliations:** Department of Animal Sciences, The Hebrew University of Jerusalem, Rehovot, Israel

**Keywords:** skeletal muscle, broiler, satellite cell, myofiber, thermal stress, myopathies

## Abstract

Myofiber formation is essentially complete at hatch, but myofiber hypertrophy increases posthatch through the assimilation of satellite cell nuclei into myofibers. Satellite cell proliferation and differentiation occur during the early growth phase, which in meat-type poultry terminates at around 8 days posthatch. Thus, any factor that affects the accumulation of satellite cells during late-term embryogenesis or early posthatch will dictate long-term muscle growth. This review will focus on the intimate relationship between thermal conditions during chick embryogenesis and the early posthatch period, and satellite cell myogenesis and pectoralis growth and development. Satellite cells are highly sensitive to temperature changes, particularly when those changes occur during crucial periods of their myogenic activity. Therefore, timing, temperature, and duration of thermal treatments have a great impact on satellite cell activity and fate, affecting muscle development and growth in the long run. Short and mild thermal manipulations during embryogenesis or thermal conditioning in the early posthatch period promote myogenic cell proliferation and differentiation, and have long-term promotive effects on muscle growth. However, chronic heat stress during the first 2 weeks of life has adverse effects on these parameters and may lead to muscle myopathies.

## Introduction

Vertebrates are born or hatch with a defined number of myofibers in the muscle. Muscle growth will then involve mainly growth in myofiber size (i.e., hypertrophy), where there is a large increase in the synthesis of contractile and regulatory proteins. In meat-type poultry (i.e., broilers), this process occurs largely from the second week of age onward and involves the accretion of nuclei. The source for these additional nuclei, at least during the early growth period, is a population of muscle progenitor cells called satellite cells (SCs; Moss and Leblond, 1971), also termed “adult myoblasts” (Hartley et al., 1992). This review will focus on the implications of environmental conditions, specifically thermal conditions, during embryogenesis and early posthatch periods, for the proliferation and differentiation of SCs, as well as for pectoralis muscle development and growth in broilers. Possible mechanisms underlying these effects will be discussed.

## Satellite Cells and Their Involvement in Postnatal Muscle Growth and Regeneration

Satellite cells were initially discovered by Mauro (1961) based on their location in the myofiber, in a niche between the basal lamina and the sarcolemma. Although originating from the somites at early stages of embryonic development (Gros et al., 2005; Relaix et al., 2005), SCs can only be distinguished at later stages; in chickens, they are present at low numbers on embryonic day 14 (E14) and become the majority at E18 (Hartley et al., 1992; Feldman and Stockdale, 1992; Yablonka-Reuveni, 1995). In contrast to the embryonic and fetal myoblasts, which undergo proliferation and terminal differentiation during the first and second waves of myogenesis in the embryo, the SCs proliferate and do not differentiate (Stockdale, 1992; reviewed in Chal and Pourquié, 2017). After birth or hatch, upon the third wave of myogenesis, SCs largely proliferate and then undergo terminal differentiation. This third wave is short; in broilers, it lasts around 8 days (Halevy et al., 2000, 2001, 2004, 2006a,b; Allouh et al., 2008). Thereafter, SC activity rapidly declines, and their numbers drop to 1–5% of total myonuclei. Satellite cells then become largely quiescent and remain in their niche in that state. They will only become active again during muscle regeneration: upon damage (e.g., injury, toxins), stress (e.g., heat or cold stress), or diseases (myopathies), SCs will be reactivated into the myogenic program, entering the cell cycle and undergoing terminal differentiation to form new myofibers (reviewed in Hawke and Garry, 2001; Zammit et al., 2006; Yablonka-Reuveni, 2011).

The muscle-specific basic helix–loop–helix family of transcription factors regulates the myogenic program of embryonic and fetal myoblasts, as well as of SCs, where they are expressed in a sequential pattern (reviewed in Naya and Olson, 1999). The paired-box containing transcription factor Pax7 plays an essential role in the formation of adult skeletal muscle (Seale et al., 2000); it is expressed by quiescent SCs and becomes highly expressed upon their activation and proliferation, then declines when the SCs undergo terminal differentiation (Halevy et al., 2004; Zammit et al., 2006).

Muscle growth and regeneration are tightly regulated by autocrine and paracrine factors, some of which are involved in SC activation and proliferation [e.g., hepatocyte growth factor (HGF), fibroblast growth factor (FGF)], whereas others are more involved in terminal differentiation and hypertrophy [e.g., insulin-like growth factor (IGF), sonic hedgehog] or inhibit these processes [e.g., transforming growth factor β (TGFβ), myostatin] (reviewed in Hawke and Garry, 2001; Halevy et al., 2006b; Yablonka-Reuveni, 2011).

Satellite cells are considered to be adult muscle stem cells due to their ability to divide in an asymmetric manner into either daughter progenitor cells that will undergo myogenic differentiation or daughter stems cells that will replenish the muscle cell reservoir (Collins et al., 2005; Shinin et al., 2006; Kuang et al., 2007; reviewed in Brack and Rando, 2012; Tierney and Sacco, 2016). Moreover, SCs, under specific environmental cues, possess the ability to transdifferentiate into other mesenchymal lineages, such as chondrogenic, adipogenic, or fibrogenic tissues (Asakura et al., 2001; Shefer et al., 2004; Brack et al., 2007; Yin et al., 2013).

## Thermoregulation in Birds

Birds are endotherms; hence, they are able to maintain their body temperature (Tb) within a narrow range. However, due to genetic selection for growth and meat production over the last decades (Havenstein et al., 2003a, b), meat-type poultry have difficulty coping with extreme environmental temperatures, especially heat stress. The consequences are adverse effects on food consumption and performance, morbidity and mortality, and inferior meat quality (Cooper and Washburn, 1998; Sandercock et al., 2001; Yahav et al., 2005; Cahaner, 2008). Among the different approaches used to avoid the harmful consequences of thermal stress and to improve the acquisition of thermotolerance are acclimation by thermal conditioning (TC) during the early postnatal period and thermal manipulations (TMs) during the incubation period (Nichelmann and Tzschentke, 2002; Tzschentke et al., 2004; Yahav, 2009). These approaches are based on the presumed occurrence of epigenetic adaptation with a lifelong impact due to changes in factors such as ambient temperature (Ta) at “critical developmental phases” of the thermoregulation system during embryogenesis and early posthatch periods (Tzschentke et al., 2004). This is reflected in a reduction in Tb, decreased thyroid and corticosterone hormone levels, and changes in the vasomotor response (Yahav, 2015). Therefore, the ability to define these “critical phases” is crucial for improving thermotolerance acquisition. Mid and late embryogenesis are good periods for TMs (Yahav, 2009), as these are the periods during which the hypothalamo–pituitary–thyroid axis, associated with thermoregulation, and the hypothalamo–pituitary–adrenal axis, associated with stress, are developing (Thommes et al., 1984). The development of the chicken brain and thermoregulation is completed during the first 10 days posthatch (Arad and Itsaki-Glucklish, 1991), making this period ideal for TC in posthatch chicks.

## Thermal Conditioning in the Early Posthatch Period Promotes SC Proliferation and Muscle Growth

The first evidence that mild TC at 37°C for 24 h promotes body weight (BW) gain in broilers emerged from experiments aimed at enhancing acclimation of broilers to hot temperatures before their marketing. Interestingly, only chicks that underwent TC on day 5 of age, but not later, had higher BW, while lower rates of morbidity and mortality were observed in all treated chicks (Yahav and Hurwitz, 1996; Yahav and Plavnik, 1999). The higher BW was accompanied by higher growth of the pectoralis muscle (De Basilio et al., 2001). Fine-tuning experiments showed that TC on day 3 posthatch is optimal for chicken acclimation to high temperatures, as well as for gains in BW and performance at marketing (Yahav and McMurtry, 2001). Satellite cell proliferative activity in chickens is at its peak on day 3 posthatch (Halevy et al., 2004, 2006b), suggesting this day to be the best time for SC activity induction by TC. Indeed, in a follow-up study, it was demonstrated that mild TC on day 3 boosts the number of SCs in the pectoralis muscle and accelerates their differentiation on later days, resulting in higher pectoral muscle growth until marketing (Halevy et al., 2001). The dramatic response of SCs to the TC was most likely due to an indirect, systemic effect rather than a direct heat effect. Analysis of different factors that might be affected by the TC revealed induction of HGF and a muscle IGF-I isotype (Halevy et al., 2001)—growth factors that are upregulated following muscle stress (Tatsumi et al., 1998; Goldspink et al., 2008). It also seemed that a reduction in triiodothyronine (T_3_) in response to the TC (Yahav and Hurwitz, 1996; Yahav, 2009) may also have an effect on the accelerated rate of SC differentiation (Ambrosio et al., 2017). Despite the promising results of TC on day 3 of age for both thermal acquisition and pectoralis muscle growth, this procedure proved to be somewhat demanding for the farmer and is therefore not widely used in the commercial rearing of broilers. Conducting TMs on chicken embryos during sensitive periods of the thyroid and adrenal axes’ development and myogenesis could provide long-lasting promotive epigenetic effects on both thermoregulation acquisition and muscle growth.

## Thermal Manipulations During Embryogenesis and Skeletal Muscle Growth

The development of the thyroid and adrenal axes in the chick embryo between E8 and E12 (Thommes et al., 1984) largely overlaps with the second wave of myogenesis, when the major part of the myofiber mass is being generated. Proliferative SC activity occurs later on, between E15 and E19, with a peak on E17 (Halevy et al., 2006a; Piestun et al., 2009b); the hypothalamo–pituitary–adrenal axis is activated during this period (Wise and Frye, 1975; Epple et al., 1997). These two periods could be ideal for TMs, to both increase thermotolerance and promote muscle growth posthatch. However, because egg incubation in the hatchery must be kept under tight temperature and humidity control, only mild changes in either timing, temperature, or duration of the TMs can be made to avoid any major stress response (Epple et al., 1997; Yahav et al., 2004a, b; Renaudeau et al., 2012). Different timings and durations of the TMs seem to have different impacts on long-term thermotolerance and pectoralis muscle growth. For example, among the wide range of temperatures and durations for TMs performed from E16 to E18, only an increase in temperature to 39.5°C for 3 h daily was optimal for the promotion of thermotolerance as well as performance (Yahav et al., 2004b; Tzschentke and Halle, 2009) and pectoralis muscle growth (Halevy et al., 2006a). However, this regime had to be reexamined for TMs on earlier embryonic days. While TMs between E8 and E10 for 3 h daily did not have any promotive effect on these parameters (Collin et al., 2007), intermittent TM for 12 h/day between E8 and E16 had a long-lasting effect on thermotolerance, BW and pectoralis muscle growth (Loyau et al., 2014) up to 70 days posthatch (Piestun et al., 2013). In another experiment, it was demonstrated that intermittent, but not continuous TM between E8 and E16, increases the embryos’ relative BW; yet, in all embryos, Tb decreased, as did thyroid hormone levels (Piestun et al., 2009a). Owing to the intermittent TM between E8 and E16, the increase in pectoralis muscle growth during embryogenesis was later reflected in increased myofiber hypertrophy (Piestun et al., 2011) and pectoral muscle growth (Piestun et al., 2013; Loyau et al., 2014) up to 35 days posthatch. The phenomenon of a long-term effect of embryonic TM on muscle growth could have several explanations. First, the immediate response to the TM was an increase in the number of myoblasts, a trend that continued posthatch, suggesting an increase in the muscle progenitor reservoir during the second and third waves of myogenesis (Piestun et al., 2015). Second, under a similar TM regime, a gene-array assay on pectoralis major muscle taken from control vs. treated embryos revealed different patterns of expression for genes involved in, among others, cell proliferation, energy metabolism, and mitochondrial function, vascularization, and muscle growth (Loyau et al., 2016). Taken together, it is concluded that TMs during periods in embryogenesis corresponding to the development of the thyroid and adrenal axes and myogenic waves result in changes in epigenetic processes, which underlie the changes in specific genes’ expression leading to long-lasting effects on thermotolerance and muscle growth.

## Negative Effects of Thermal Stress on SC Myogenesis in Posthatch Chicks

The early posthatch period is critical for muscle growth; during this period, SCs complete their proliferation and undergo terminal differentiation and fusion to myofibers. Therefore, changes in nutrition (Halevy et al., 2000; Mozdziak et al., 2002; Bigot et al., 2003; Kornasio et al., 2011; Velleman et al., 2014; Powell et al., 2014, 2016) or environmental conditions (Halevy et al., 1998, 2001; Liu et al., 2010; Hadad et al., 2014a) during this period largely affect these processes, resulting in long-term effects on muscle growth. As already noted, TC on day 3 of age is beneficial for SC proliferation and differentiation (Halevy et al., 2001). However, prolonged thermal stress during the first 2 weeks posthatch had the opposite effect on these cells. Cell proliferation and numbers declined along with myofiber hypertrophy (Hadad et al., 2014a; Piestun et al., 2017; Patael et al., 2019), all of which had short- and long-term adverse effects on BW and pectoralis muscle weight (Hadad et al., 2014a, b; Patael et al., 2019). Interestingly, these adverse effects were minimal or non-existent under mild heat stress, where the temperature was only 2°C higher than commercial production temperatures. Moreover, in chicks grown under mild cold conditions, the proliferation of SCs exceeded that in the other groups (Patael et al., 2019). Taken together, it can be concluded that the Ta under which chicks are reared early posthatch is a critical factor influencing the immediate activity of the muscle progenitor cells and muscle hypertrophy at later ages.

Detailed studies on cultured SCs from broilers (Harding et al., 2015, 2016) and turkeys (Clark et al., 2016, 2017) revealed that the pectoralis muscle, in particular, is highly sensitive to temperature changes. Yet, a decline in SCs did not result from increased apoptosis in either cultured cells (Harding et al., 2015) or the treated chicks (Patael et al., 2019). A surprising finding was that the heat stress caused dramatic changes in SC fate toward adipogenesis and in muscle structure toward myopathy. Accumulation of lipid droplets was noticed shortly upon heat stress of cultured SCs (Harding et al., 2015; Clark et al., 2017), in SCs derived from heat-treated chicks (Piestun et al., 2017), and later, as the cells underwent differentiation, in myotubes and myofibers. The lipid accumulation was due to the induction of adipogenic gene and protein expression in the SCs (Harding et al., 2015; Clark et al., 2017; Piestun et al., 2017; Patael et al., 2019), suggesting transdifferentiation of these cells to the adipogenic lineage. Interestingly, these SCs did not seem to lose their myogenic characteristics completely, and along with the fat deposition, they underwent terminal differentiation to myotubes and fusion to myofibers. It may well be that, although the myogenic phenotype remains, these specific myofibers lose their full ability to contract, thus rendering the muscle less functional and contributing to its myopathic appearance.

## Thermal Stress in the Early Posthatch Period and Pectoral Muscle Myopathies

**FIGURE 1:**
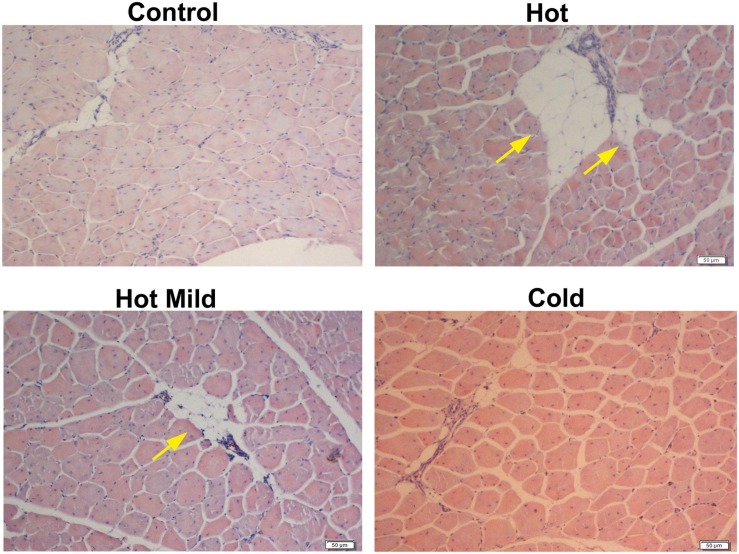
Morphological structure of pectoralis muscle in cross-sections derived from control and treated chicks on day 35 of age and stained with hematoxylin–eosin. Meat-type chick (Cobb strain) groups were reared in temperature-controlled rooms under various Ta regimes beginning on day 1 and gradually reduced until day 13, as follows: control, commercial temperature, 33–27°C; hot, 39–33°C; mild hot, 35–30°C; cold, 29–27°C. From day 14 onward, Ta was similar to all groups. Note the relatively smaller myofiber diameters in the hot group as compared to the other groups, and the large areas of fat deposition in the hot and hot mild groups (yellow arrows). Bar, 50 μm. Adapted from Patael et al. (2019), used with permission.

Chronic heat stress, even when mild, during the first 2 weeks posthatch causes long-term adverse effects on the pectoralis muscle. The pectoralis muscle of the heat-stressed chicken presents large amounts of collagen deposition (i.e., fibrotic tissue) between the myofibers and large areas of fat droplets (Figure 1; Patael et al., 2019), all of which are typical features of myopathies (Kuttappan et al., 2016; Sihvo et al., 2014, 2017; Velleman et al., 2018). The various myopathies in poultry, such as white striping, wooden breast, and spaghetti meat, are thought to result from genetic selection for BW and, in particular, breast meat production, and inaccurate or suboptimal nutrition and/or management (Branciari et al., 2009; Kuttappan et al., 2012, 2016; Petracci et al., 2013; Velleman and Clark, 2015; Tonniges et al., 2019). These myopathies have a severe economic impact on the poultry industry due to a sheer loss in meat quantity and quality (Alnahhas et al., 2016; Kuttappan et al., 2016; Baldi et al., 2018). The direct metabolic and physiological causes underlying these myopathies are not fully understood. They could be due, at least in part, to extensive genetic selection for BW or pectoral muscle growth, but not for supportive tissues (e.g., cardiovascular system), leading to lack of vascularization mainly in the pectoralis muscle and, hence, lack of oxygen supply and energy storage (Alnahhas et al., 2016; Malila et al., 2019). A very recent study reported a direct association between dysregulation of lipid metabolism and the development of wooden breast (Papah and Abasht, 2019). Thermal stress, especially during the early growth period, probably further aggravates this situation, leading to myodegeneration, expansion of the muscle’s interstitial fibro-adipogenic progenitors (Contreras et al., 2016), and/or recruitment of myofibroblasts and adipogenic cells from nearby tissues, resulting in increased fibrotic tissue and fat deposition between the myofibers (Bailey et al., 2015). This is in parallel to the reduction in the number of SCs and their changes in fate, which may lead to ineffective myofiber function (Harding et al., 2015; Piestun et al., 2017; Patael et al., 2019). In contrast, featherless broilers subjected to cold stress during the first week posthatch developed a better vascular system and had a higher number of muscle cell progenitors and better muscle hypertrophy than feathered broilers (Hadad et al., 2014a, b). In addition, broilers that were kept under colder temperatures during the first 2 weeks posthatch had hardly any signs of collagen or fat deposition in the pectoralis muscle (Figure 1; Patael et al., 2019). Taken together, the evidence suggests that thermal stress should be minimized in modern broilers that have high rates of metabolism and heat production, on the one hand, and a shortage of blood supply on the other.

## Conclusion

Changes in Ta have a pronounced impact on muscle growth, as well as on thermal acclimation in birds in general and in fast growing poultry in particular. These changes are observed already during embryonic development and into the posthatch period. During periods when they are highly active, SCs are very sensitive to temperature changes; these changes affect their proliferation and differentiation activities, as well as their fate, with long-term effects on muscle growth and structure. While short and mild heat stress promotes SC activity and muscle growth, more chronic heat stress has severe consequences, even resulting in muscle myopathies. Therefore, the duration and degree of the change in Ta, as well as its timing during the embryonic and posthatch periods, are of the utmost importance in determining the development and growth of muscle in poultry.

## Author Contributions

The author confirms being the sole contributor to this manuscript and approved it for publication. OH conceptualized the manuscript, reviewed the literature, and wrote the manuscript in its entirety.

## Conflict of Interest

The author declares that the research was conducted in the absence of any commercial or financial relationships that could be construed as a potential conflict of interest.

## Abbreviations

BW: body weight
SC: satellite cell
Ta: ambient temperature
Tb: body temperature
TC: thermal conditioning
TM: thermal manipulation.
